# Dependence and Guidance Receptors—DCC and Neogenin—In Partial EMT and the Actions of Serine Proteases

**DOI:** 10.3389/fonc.2020.00094

**Published:** 2020-02-04

**Authors:** Trevor W. Stone

**Affiliations:** NDORMS, University of Oxford, Oxford, United Kingdom

**Keywords:** DCC, neogenin, chymotrypsin, subtilisin, serine proteases

## Abstract

The Epithelial-Mesenchymal Transition (EMT) is an important concept in understanding the processes of oncogenesis, especially with respect to the relationship between cell proliferation and metastatic properties such as spontaneous cell motility, chemotaxic migration and tissue invasion. EMT is now recognized as a more complex phenomenon than an all-or-nothing event, in which different components of the EMT may have distinct roles in the physio-pathological regulation of cell function and which may in turn depend on differential interactions with cell constituents and metabolic products. This mini-review summarizes recent work on the induction of cancer properties in parallel with the presence of EMT activities in the presence of serine proteases, with the focus on those tumor suppressors known as “dependence” receptors such as neogenin and Deleted in Colorectal Cancer (DCC). It is concluded that various forms of partial EMT should be given more detailed investigation and consideration as the results could have valuable implications for the development of disease-specific and patient-specific therapies.

## Introduction

The concept of Epithelial-Mesenchymal Transition (EMT) was founded on changes in adhesion molecules associated with proliferation, cell dis-association, de-differentiation to a more mesenchymal stem cell character and the establishment of a motile, potentially invasive phenotype (1). EMT was thought necessary to initiate cell departure from a home tissue, a phenomenon seen in embryogenesis and in the metastatic spread of cancers. Recent studies have begun to define “partial” forms of EMT while others propose that EMT is more concerned with the end-stages of migration and invasion, with the reverse process of MET being at least as important as EMT (2, 3). EMT may be critical to the physical stabilization of cells in a metastatic location and in the development of increased resistance to chemotherapeutic drugs. The role of EMT in carcinogenesis may generate new sets of biomarkers for early metastasis detection and novel targets for dedicated drug development.

The complexity of molecular changes associated with EMT was responsible for the proposal of sub-types of EMT, or “partial” EMT, and the possibility that different molecular profiles may relate to the activity and aggressiveness of tumors. Partial EMTs may take many forms, based on different molecular profiles which characterizes different cell types, tissue locations and pathological conditions. The concept predicts that partial EMTs may provide a key for the generation of individual, tissue and disease specificity necessary for developing successful personalized therapy of cancers and other disorders. Defining the factors which induce partial EMTs will be essential if these relationships are to be exploited therapeutically. This mini-review surveys evidence for a role, in these events, of Dependence Receptors and their modulation by serine proteases.

## Dependence and Guidance Receptors (DGRs)

**Figure 1:**
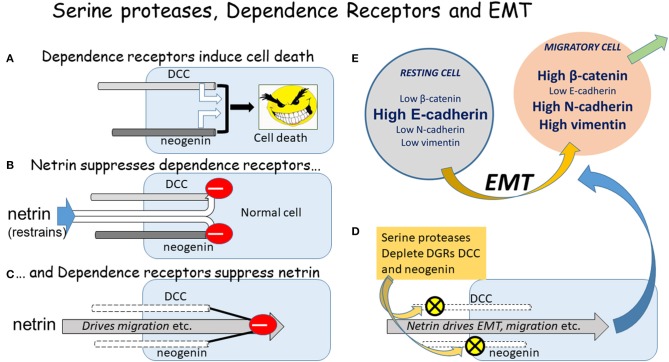
The DGRs, serine proteases, and EMT. A diagrammatic summary of the relationship between the DGRs DCC and neogenin, serine proteases, and the initiation of EMT. **(A)** If un-restrained by ligand binding, the DGRs promote cell death. **(B)** The primary ligands of DCC and neogenin are the netrin proteins, which restrain their ability to induce apoptosis. **(C)** Conversely the DGRs exert a feedback restraint on the netrins which tend to drive cells into an aggressive, pro-migratory mode via the induction of EMT. **(D)** The serine proteases chymotrypsin and subtilisin deplete DCC and neogenin from cells, allowing netrin to drive EMT and migration. **(E)** The classical EMT involves changes in the absolute and relative levels of proteins such as those indicated, but the magnitude and direction of change depends on the cell type, inducing stimulus, local microenvironment, and physio-pathological status, yielding a plethora of possible combinations of change which could represent highly selective therapeutic targets.

The Dependence Receptors include Deleted in Colorectal Carcinoma (DCC) and the structurally related molecule neogenin (49% amino acid identity) (4). The term “dependence” receptors indicates that cell viability depends on ligation of the receptors by extracellular ligands such as the netrin family of extracellular proteins (ligands for DCC and neogenin) (5–8) or the Repulsive Guidance Molecules (RGMs; ligands for neogenin only). When occupied the receptors maintain cell survival, restraining cells from initiating apoptotic death when deprived of their ligands (9, 10) (Figure 1). These same receptors play crucial “guidance” roles, influencing the direction and rate of cell movement and the direction and size of cell outgrowths such as lammellipodia. These actions are seen in embryonic development when the dependence receptors guide progenitor cells to their eventual locations (11, 12), and in the developing and adult nervous system where they influence axonal or dendritic growth as well as the positioning of newly formed neurons (13–16), synaptic contacts and neural plasticity (17, 18). In view of this functional duality, the receptors will be referred to as Dependence and Guidance Receptors (DGRs).

Also of relevance to EMT is the unco-ordinated-5 (unc5) family of DGRs—homologs of the uncoordinated locomotion genes in *Caenorhabditis elegans*—which also bind netrins as ligands (19). The binding of netrin-1 to DCC normally induces growth toward the focus of netrin production (20). If DCC is absent and netrin binds to unc5, then the result is repulsion: cells are directed away from the site of highest netrin concentration. Interaction between the intracellular domains of DCC and unc5 converts DCC attraction to repulsion (21). It would seem very likely that the migratory drive, direction and eventual target re-location of cells bearing these DGRs will depend on the ratios of attractant and repellent molecular states and may be critical in the establishment of metastases.

### Relationships Between DGRs and EMT

Cell dependence on DGRs may have evolved in response to the development of multicellularity and distinct tissues. A cell could develop a genetic mutation spontaneously or induced by an infective organism, toxic compounds or physical trauma, which reduced intercellular adhesion—a necessary early stage of cell dis-association needed for EMT. A cell which becomes detached from the home tissue will then enter the extracellular medium as a free-living, migratory cell. The potential for early stage cancerous cells to establish metastatic colonies is clear. However, after leaving the home tissue the fall in ligand concentration for the cell's DGRs induces the unbound receptors to promote caspase-mediated apoptotic death (anoikis) (9, 22), a result which has been considered a safety mechanism to minimize or prevent the development of metastases.

In some cases, cells could become established at sites where a sufficient concentration of netrin isoforms allows them to create a new micro-colony (metastasis), since many tissues are able to synthesize and secrete these proteins (23–26). This may require the migration of a minimum number of cells which, once established, could produce enough ligand to maintain the metastatic colony. If this hypothesis were correct, it would suggest the need to inhibit tumor development as early as possible to prevent metastatic deposits achieving a size at which their ligand production offers continuing survival.

We have shown that DGR function may be regulated by chymotryptic serine proteases which deplete cells of their DGRs, causing increased cell migration (27), actions that may contribute to dietary effects on cancer incidence (28, 29). Subsequently, the effects of this DGR loss on the induction and progression of EMT were considered to identify any relationship between them, as discussed in the following sections in which the major EMT proteins E-cadherin, N-cadherin, β-catenin and vimentin were investigated (Figure 1) (30).

#### E-Cadherin

E-cadherin is localized primarily to cell membranes where it is involved in the formation of intercellular *adherens* junctions. It is considered a marker for resting, physiologically stable epithelial cells and its levels fall as cells de-differentiate before adopting the metabolic and morphological characteristics of migratory cells entering the EMT (31–35). In contrast, N-cadherin expression usually increases with the approaching onset of EMT and migration (36). The depletion of E-cadherin has been associated with carcinogenesis and metastasis formation (34, 37–39) and there is moderate expression in potentially metastatic cells. It has been shown that high levels of E-cadherin occur in metastases but it is absent from many primary tumors (40, 41).

#### β-Catenin

E-cadherin is usually associated with β-catenin and loss of the former releases β-catenin to enter the cytosol and nucleus where, as part of the wingless (Wnt) pathway, it regulates gene expression to influence proliferation and migration (31, 38, 41–54). It has been considered as the most significant protein characterizing EMT (55–60). However, β-catenin activation has also been linked to an inhibition of cell growth (61) and in some cases has little effect on cell behavior (62) while around 30% of mammary cancer cells had little or no β-catenin, raising questions about the extent and timing of its involvement in tumor development (63). β-catenin may have other functions in the cell independent of proliferation or migration, including the maintenance of epithelial structure and differentiation (51).

#### Vimentin

Vimentin is a microfilament protein involved in cytoskeletal function. As EMT develops, vimentin levels increase with cytoskeletal reorganization required for migration (64–68). In the absence of vimentin, migration is significantly reduced (69) as the E-cadherin/vimentin ratio may determine the switch from low to high invasion capacity. Transforming Growth Factor-β, a major regulator of EMT, may regulate vimentin expression and the decrease in E-cadherin (70, 71). However, vimentin is not always increased in migratory cells (72) and simple changes in incubation conditions, such as reducing the proportion of serum, can induce vimentin expression (27, 30). Vimentin expression might therefore be linked to the differentiation state of cells rather than their migratory status or capacity (73).

### Serine Proteases, DGRs, and Partial EMT

Contradictory results have also been described when cells were exposed to serine proteases such as mammalian chymotrypsin or the bacterial chymotryptic enzyme subtilisin (27, 30). These proteases reduced expression of DCC and neogenin, with changes in the EMT proteins that were not consistent with normal views on their relationship to EMT. In particular, while levels of E-cadherin were reduced with the onset of migration, β-catenin levels were unaffected or substantially reduced, observations also obtained by other groups (45, 74). Vimentin expression was increased, as would be predicted for a compound involved in cytoskeletal adaptations to a change in differentiation state, dissociation and migration.

This pattern of protein changes associated with a loss of DGRs is clearly different from classical descriptions of EMT, suggesting that DGR loss should be added to the list of phenomena able to induce a “partial” EMT. Similar results have been obtained by silencing the neogenin gene in the absence of other primary modifications (75). This selective loss of neogenin also provoked changes in EMT proteins which were not fully consistent with conventional views of EMT leading to the conclusion that (from genetic and morphological data), a “partial EMT” was produced. Importantly, the results not only extend our own work but also confirm that the serine protease effects were probably produced solely by actions involving DGRs.

With the growing recognition of partial EMTs (76–83) some initiating factors are being identified including phosphatidyl-inositol-3-kinase (84), TGF-β (85–87), galectins (88), or altered expression of PTEN (89). Some groups define partial EMT with respect to mismatch between cell protein profile and phenotypic behavior (90) while others are attempting to define RNA fragments which regulate protein expression. These include miR-376a, miR-381, and miR-128 for which evidence indicates a significant relationship to tumor diagnosis and progression (91).

### DGRs, EMT, and Differentiation

DCC expression declines as tumors advance and cells become more de-differentiated (92, 93). This would be consistent with the suggestion that EMT is a reflection of the differentiation status of cells and might explain the changes in vimentin expression induced by reduced serum-containing media (30, 73). It would account for the loss, rather than increase in β-catenin expression since β-catenin levels fall in cells differentiated by retinoic acid (94). It is also consistent with the need for de-differentiation for cell migration (95).

DCC transfection into cells inhibits proliferation and migration in some cell populations (96–99) but it remains uncertain whether the expression of DGRs such as DCC and neogenin (100) are primary inducers of cancerous change or are secondary contributors to oncogenesis (45, 101–105). The possible association between DCC and the progress of EMT rather than its initiation would be consistent with several studies (103, 106). Perhaps viewing cell differentiation as a key to this uncertainty, rather than the EMT or any individual component of it, may help to resolve the issue (93, 107, 108). DCC is relatively abundant in fully differentiated cells but declines in the early stages of carcinogenesis (109), falling further with advancing tumor stage and reduced patient survival (13, 99, 103, 105, 106, 108, 110–112). Conversely, but very importantly, differentiation induced by a range of factors may only be possible in the presence of DCC (111, 113).

### EMT or MET?

A modified view on the link between EMT and differentiation is that EMT may be associated with the termination of cell migration rather than with its initiation. Post-migratory cells undergo changes of EMT proteins which are the reverse of classical EMT changes and which may be crucial for cell re-settlement in a metastatic deposit (114). Interestingly, the loss of protein *frazzled* (a homolog of DCC) inhibits MET suggesting that DCC may normally drive the non-migratory state of cells by promoting MET, while its progressive decline with tumor growth eventually leads to an apparent EMT and metastatic migration. The netrin ligation of DCC induces a loss of cadherin and the initiation of EMT, raising the possibility that in the absence of netrin, a re-expression of DCC might enhance E-cadherin expression and MET, consolidating metastasis formation (52). Of course, a migrating cell will be identified as a “foreign” cell and will normally be removed by the immune system unless it can re-express DCC. Cells incapable of expressing DCC would remain susceptible to removal unless they migrate to areas sufficiently safely hidden from immune system cells, or where the local concentration of ligand is sufficient to overcome immune attack. These ideas would be consistent with suggestions that DCC increases in later stages of colony formation, when it promotes cell differentiation (with increased E-cadherin). The explanation also accounts for DCC-expressing cells being sensitive to anti-cancer drugs, whereas truly metastatic cells are relatively resistant (2, 3, 115, 116).

## Conclusions

These arguments represent a highly simplified view of molecular events underlying changes in cell properties and behavior. In addition to DGRs, other important factors in EMT include growth factors (especially Transforming Growth Factor-β), oxidative stress, micro-RNAs and immune system cells (macrophages, neutrophils) (117–119). Since neutrophils generate large quantities of chymotryptic serine proteases, they are likely sources of the proteases discussed in this mini-review.

The term “partial EMT” conveys the concept that cells are exhibiting changes which are partially characteristic of epithelial cells and partially typical of mesenchymal cells. On the existing evidence, however, there is likely to be a spectrum of such states, probably dependent on the cell type, preceding mutagenic events and the final EMT-initiating stimulus (119). It may be that these proteins do not fully describe the intermediate states and the temporal sequence of cell properties, or the protein changes may represent a non-canonical EMT-like process or an independent mechanism for migration. These alternative possibilities might be more consistent with the suggestion that DCC is more functionally related to the stabilization and differentiation aspects of EMT or MET than to migration and metastasis. Indeed, vimentin was the only EMT protein whose expression increased with the increased migration generated by serine proteases. Other descriptions of mixed EMT protein changes include the “transition state” in brain metastases exhibiting atypical expression of E-cadherin and vimentin (32). Thus, vimentin may be a more selective component to study in relation to EMT (120).

Several transcription factors once thought to be essential for EMT are dispensible since, for example, Twist and Snail do not suppress cell invasiveness (2). In addition, EMT proteins may have actions which are unrelated to EMT (121). Overall, however, EMT may contribute to aspects of cell function with changes in protein expression which respond differently to changes in cell status. This could yield cells with a variety of partial “EMT signatures” unique to that cell population, disease and pathology. It is difficult to assess whether EMT is more usefully viewed in terms of the “partial” concept of organizational discontinuity rather than as a continuum of activity (78, 122), since even a continuum of molecular changes may generate discontinuous functional events with different thresholds for switching cell behavior with changes in protein signature. This will be difficult to clarify if those thresholds involve critical ratios of several compounds which individually appear to vary continuously. Nevertheless, developing these could facilitate the generation of new anti-cancer therapies with a high degree of specificity and safety for individual patients, cancer type and stage, depending on the cell signature involved. This may be especially important since partial EMTs may be associated with more aggressive and chemoresistant tumors (123–126) although the contrary view has been expressed, emphasizing the need for further work (127).

## Author Contributions

TS performed the literature search and wrote the mini-review.

### Conflict of Interest

The author declares that the research was conducted in the absence of any commercial or financial relationships that could be construed as a potential conflict of interest.
